# The neural connections of oxytocin-mediated parental behavior in male mice

**DOI:** 10.3389/fnmol.2023.1091139

**Published:** 2023-02-24

**Authors:** Zhichao Chen, Qian Wang, Xiumin Xue, Zhihui Huang, Yongjie Wang

**Affiliations:** ^1^School of Pharmacy, Hangzhou Normal University, Hangzhou, Zhejiang, China; ^2^Key Laboratory of Elemene Class Anti-Cancer Chinese Medicines, Hangzhou Normal University, Hangzhou, Zhejiang, China; ^3^Engineering Laboratory of Development and Application of Traditional Chinese Medicines, Hangzhou Normal University, Hangzhou, Zhejiang, China; ^4^Collaborative Innovation Center of Traditional Chinese Medicines of Zhejiang Province, Hangzhou Normal University, Hangzhou, Zhejiang, China

**Keywords:** oxytocin neurons, oxytocin ligand, virgin males, parental caregiving behaviors, behavioral plasticity

## 1. Introduction

The adult brain can flexibly adapt behaviors to specific life-stage demands, and a classical example of this plasticity of neural circuits is the emergence of infant-rearing behavior in male mice. Both physically and mentally, the care that fathers provide is necessary for the growth of pups (Svetaz et al., 2014). Fathers' influence on pups can be subtle and far-reaching (Schorr et al., 2021; Scott et al., 2021). When male mice are sexually naive, they usually ignore or even attack cubs. In contrast, after becoming sexually mature, they will display caring behavior toward their own young. However, it remains unclear how caregiving behavior plasticity is implemented at the level of neural connections.

A recent study reported that this significant alteration might be due to the effect of oxytocin (OT) on mammals (Froemke and Young, 2021). OT is a neuropeptide elaborated by the hypothalamic paraventricular (PVN) and supraoptic (SON) nuclei. It is mainly involved in the promotion of childbirth and milk ejection (Uvnas-Moberg et al., 2019; Perkinson et al., 2021). Moreover, the link between OT and social behavior has been extensively studied in recent years (Bosch and Young, 2018). Research has revealed that OT can increase mutual trust among humans (Strauss et al., 2019) and reduce the risk of mental illnesses such as anxiety and depression (Naja and Aoun, 2017). In addition, increased OT secretion can reduce aggressive behavior of male mice (Steinman and Trainor, 2017). Exploring the specific mechanism of the intrinsic neural circuit between OT secretion and caring behavior in male mice is of great significance.

## 2. OT is indispensable for the parental caregiving behaviors of male mice

**Figure 1:**
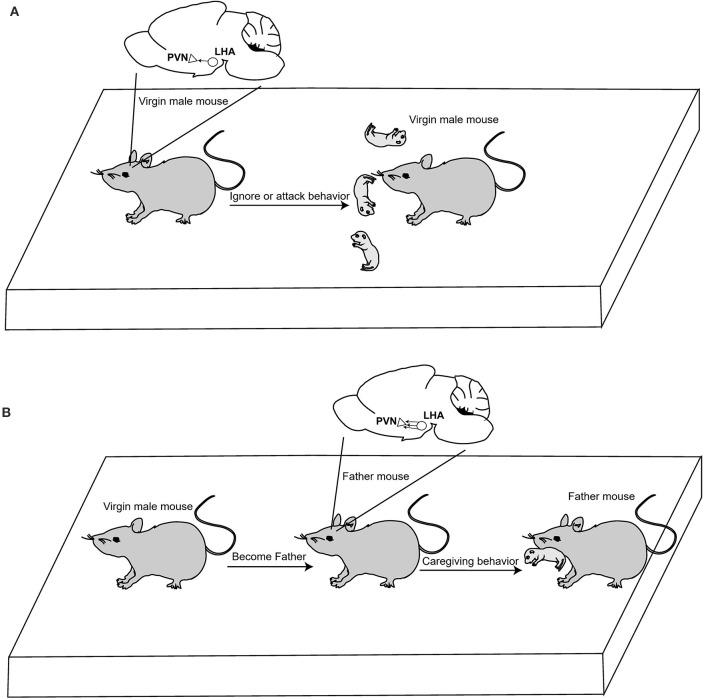
OT neuron activation facilitates caregiving behavior in virgin male mice. **(A)** In normal conditions, virgin male mice attack and ignore pups; they do not display parental caregiving behavior. **(B)** Neural connections originating from the LHA to the PVN OT neurons were drastically strengthened when male mouse became father. Activation of OT neurons in the PVN can induce caregiving behavior.

Recently, Kazunari Miyamichi's team from Japan's RIKEN Research Institute published an article in *Neuron* that revealed the neural circuit plasticity mechanism of OT in regulating the parent-child behavior of male mice (Inada et al., 2022). The authors used chemogenetic viruses to activate OT neurons in the PVN of virgin male mice and found that they exhibited parenting behaviors and reduced aggression toward pups. In addition, viral tracer results showed that excitatory connections from the lateral hypothalamus (LHA) to OT neurons in PVN were enhanced when virgin male mice became fathers. These connections are functionally relevant, as their activation can induce parental behavior in virgin male mice (Figure 1). The adult brain can flexibly adapt behaviors to specific life-stage demands. These discoveries help providing scientific support for the mitigation of mental health conditions in children that are caused by a lack of parental caregiving behaviors. However, it remains unclear how caregiving behavior plasticity is implemented at the level of neural connections.

First, to explore the relationship between parenting behavior and OT in male mice, they used CRISPR-Cas9-mediated gene editing technology and Cre-expressed adeno-associated virus to conditionally knock out OT (OT^−/−^) in the PVN. Then, they established a behavioral assay and applied it to wild-type (OT^+/+^) and OT^−/−^ mice to assess caregiving behaviors; three unfamiliar pups from different families were put into three cages, and a father mouse that was unrelated to these pups was allowed to interact with them freely. While the OT^+/+^ mice displayed caring behavior, a large percentage of the OT^−/−^ male mice ignored the young mice. This result indicates that OT neurons in the PVN are necessary to exert parental behaviors in fathers. Further examination of expectant fathers illustrated that the activity of OT neurons is essential for the first appearance of paternal caregiving behaviors.

Does OT have the same effect on virgin male mice? To address this, the authors used the chemogenetic virus hM3Dq-mCherry to activate OT neurons in the PVN of virgin male mice. They found that these mice exhibited more pup-care-related behaviors and less aggression. Moreover, Ucn3+ neuronal (related to infanticide) activities in the perifornical area (peFA) were inhibited, whereas the neural activities of Calcr+ neurons (a center for parental behaviors) in the medial part of the MPN (MPNm) were elevated. These outcomes illustrate that OT neurons could regulate the limbic neural populations related to parental and infanticidal behaviors in virgin male mice.

Moreover, OT neurons can release non-OT neurotransmitters and neuropeptides. To identify their role in parenting behavior in the PVN, they used hM3Dq-myc driven by an OT promoter (OTp) to activate OT neurons in virgin male mice. After injection of clozapine N-oxide, which can lead to the release of neurotransmitters or neuropeptides other than OT in OT^−/−^ mice, increased caregiving behaviors were displayed by the OT^+/+^ mice but not the OT^−/−^ virgin male mice. Nevertheless, pup-directed attacks between these two genotypes were suppressed after the injection. Taken together, these data suggest that, compared to the significant promotion from OT, other neurotransmitters are also conducive to caregiving behaviors.

To further investigate the change in neural connections of paternal behavior, the authors concentrated their work on PVN OT neurons. Using rabies virus-based retrograde trans-synaptic tracing, OT neurons in the fathers displayed more input from the LHA and MPNm without a change in the number of neurons compared to virgin males. Interestingly, the increased inputs observed 5 days after the birth of the pups returned to a level similar to that of virgin males after 5 weeks of isolation, revealing that the enhanced connection was temporary and reversible. Moreover, histochemical methods and further analysis of cell-type-specific marker genes demonstrated that the inputs to PVN OT neurons were mostly excitatory neurons from the LHA and MPNm, especially melanin-concentrating hormone-producing excitatory neurons in the LHA.

To examine the electrophysiological properties of excitatory inputs to OT neurons, they injected the AAV-OTp-mCherry virus into the PVN and AAV-FLEx-ChR2 (H134R) virus into the LHA, MPNm, and dorsomedial hypothalamus (DMH) of *vesicular glutamate transporter type 2* (*vGluT2*)-Cre mice. Consistent with the trans-synaptic tracing results, the excitatory postsynaptic currents evoked by the optogenetic stimulation of LHA and MPNm inputs were slightly, but not statistically significantly, larger in the fathers. However, the response to the DMH stimulation remained unchanged. Furthermore, optogenetic activation of excitatory neurons in the LHA evoked more spikes in PVN OT neurons, which would be even more when MPNm was activated concomitantly. These results demonstrate that the enhanced excitatory connectivity from the LHA to OT neurons is associated with life-stage transition.

Finally, what was the specific relationship between excitatory connections from the LHA to OT neurons and parental behavior? Through the use of *in situ* staining, they found that fathers who interacted with pups expressed *c-fos* at a higher ratio in the LHA than those who were not exposed to pups. Notably, most *c-fos*+ neurons were *vGluT2*-positive and Pmch-expressing excitatory neurons. They focused on the functional contributions of this connection. Chemogenetic inhibition of *vGluT2*+ LHA neurons only slightly increased aggressive behaviors toward the pups in virgin male mice. Conversely, targeted hM3Dq-myc in the *vGluT2*+ LHA neurons of OT^+/+^ and OT^−/−^ virgin males showed a reduction in aggression toward the pups of virgin males without evoking caregiving behaviors. More importantly, OT^+/+^ virgin males were significantly less aggressive than their OT^−/−^ counterparts. Taken together, OT release mediated by excitatory LHA neurons provokes the parental behaviors of fathers by suppressing infanticide.

## 3. Discussion

Fathers play a unique role in the growth of their children (Volling et al., 2019). The specific mechanisms of neural connections in male animals' parenting behaviors have been extensively explored for decades but remain unclear.

Miyamichi et al. first established a behavioral assay to test the paternal caregiving behaviors of male mice, enabling the analyses between behavior and basic neuroscience to be more visual and precise. In addition, they chose OT, a neuropeptide that modulates numerous brain functions and utilized rabies-virus-mediated unbiased screening and cell-type analysis to comprehend the neural connections of OT. Interestingly, their study revealed that the structural plasticity of adults might be greater than expected. Furthermore, their research findings may not be just limited to paternal caregiving behavior but may also be applied to other life-stage transitions or even transient behavior changes.

OT, acts in the brain as a non-canonical neurotransmitter or neuromodulator, has been long known to shape behavior in rodents (Cherepanov et al., 2021; Zhang et al., 2021). OT originating from the PVN modulates various social behaviors, such as social recognition, fear memory, parental behavior (Hasan et al., 2019). Disorders in OT secretion involve in many psychiatric disorders including depression, anxiety, schizophrenia, and autism spectrum disorders (Lefevre et al., 2021). Notably, OT can have different modulatory effects on the same function under different conditions. Such divergence may derive from different neural connections (Wang et al., 2022). Recently, Scott et al. (2015) showed that tyrosine hydroxylase (TH)-expressing neurons in the anteroventral periventricular nucleus of the rodent hypothalamus are related to parental behavior. TH^+^ cells have been shown to relay monosynaptic inputs to oxytocin expressing neurons and are thought to regulate oxytocin secretion. TH^+^ anteroventral periventricular nucleus (AVPV) neurons can facilitate OT release from OT^+^ PVN neurons into central nervous system and blood, leading to parental behavior. Although OT modulation in rodents' behaviors have been extensively studied, little is known on its mechanism in the regulation of parental caregiving behaviors of virgin male mice.

Lack of parental caregiving behaviors can cause mental diseases, such as social behavior, dysfunction, depression, anxiety and so on. The oxytocinergic systems in the CNS are associated with or influence processes implicated in depressive and anxiety disorders as well as those underlying stress, making OT potentially relevant to the development, maintenance, and treatment of these conditions. OT has been shown to exert anxiolytic and antidepressant effects (Slattery and Neumann, 2010; MacDonald and Feifel, 2014). Acute and chronic administration of intranasal OT have been extensively utilized in both animal models and human preclinical and clinical studies to treat various related mental diseases (Rae et al., 2022).

However, several issues related to this research need to be explored further. For instance, what is upstream of the LHA and downstream of PVN OT neurons? Why and how is the connection between the LHA and PVN OT neurons strengthened after virgin male mice become fathers? What is the specific molecular mechanism? Could both inhibition of LHA and peFA suppress pup-directed attack? The role of non-OT neurotransmitters/neuropeptides in this function remains unclear. Besides, some paternal behaviors that indirectly contribute to offspring fitness, such as provisioning and the connection between territorial defense and OT, have not been well-elucidated.

Intranucleus OT release from PVN neurons and into the bloodstream from the nerve terminals of this nucleus in the posterior pituitary (Eliava et al., 2016). OT neurons from PVN project centrally to forebrain regions can modulate neurocircuitry related to learning and memory, anxiety, fear, social approach and reward to treat diseases (Stoop, 2014). OT delivering *via* the intranasal (IN) is a major clinical drug delivery route, which is a more easier, more efficient administration way and can protect the body from systemic toxicity (MacDonald et al., 2011). However, OT's short half-life becomes an obstacle to its treatment of diseases. The application of nano-based delivery system not only improves the penetration of OT inside brain but also increases its half-life by the application of encapsulation and extends release (Al-Suhaimi et al., 2021).

In conclusion, Miyamichi et al. demonstrated that PVN hypothalamic oxytocin neurons and OT ligands are key regulators of parental caregiving behaviors in male mice. The plasticity of the hypothalamic neural connections is related to life stages, long distances, and specific cell types. These considerable discoveries help to provide scientific support for the mitigation of mental health conditions in children that are caused by a lack of parental caregiving behaviors and present a pattern for investigating other behavioral changes.

## Author contributions

ZC, QW, XX, ZH, and YW wrote and edited the manuscript. All authors have contributed to the manuscript and approved the submitted version.
